# The Prospects of Non-EEG Seizure Detection Devices in Dogs

**DOI:** 10.3389/fvets.2022.896030

**Published:** 2022-05-23

**Authors:** Jos Bongers, Rodrigo Gutierrez-Quintana, Catherine Elizabeth Stalin

**Affiliations:** Neurology and Neurosurgery Service, The School of Veterinary Medicine, College of Medicine, Veterinary Medicine and Life Sciences, University of Glasgow, Glasgow, United Kingdom

**Keywords:** wearable, technology, canine, epilepsy, review, seizure detection, dog

## Abstract

The unpredictable nature of seizures is challenging for caregivers of epileptic dogs, which calls the need for other management strategies such as seizure detection devices. Seizure detection devices are systems that rely on non-electroencephalographic (non-EEG) ictal changes, designed to detect seizures. The aim for its use in dogs would be to provide owners with a more complete history of their dog's seizures and to help install prompt (and potentially life-saving) intervention. Although seizure detection via wearable intracranial EEG recordings is associated with a higher sensitivity in humans, there is robust evidence for reliable detection of generalized tonic-clonic seizures (GTCS) using non-EEG devices. Promising non-EEG changes described in epileptic humans, include heart rate variability (HRV), accelerometry (ACM), electrodermal activity (EDA), and electromyography (EMG). Their sensitivity and false detection rate to detect seizures vary, however direct comparison of studies is nearly impossible, as there are many differences in study design and standards for testing. A way to improve sensitivity and decrease false-positive alarms is to combine the different parameters thereby profiting from the strengths of each one. Given the challenges of using EEG in veterinary clinical practice, non-EEG ictal changes could be a promising alternative to monitor seizures more objectively. This review summarizes various seizure detection devices described in the human literature, discusses their potential use and limitations in veterinary medicine and describes what is currently known in the veterinary literature.

## Introduction

A reduction in seizures is typically used as the outcome measure in research papers in canine epilepsy largely focussing on anticonvulsant medication (1–4). However, there is increasing recognition that other factors influence outcome such as adverse effects of medication and the effect that caring for a dog with epilepsy has on the owner. Several studies have investigated the quality of life in dogs with idiopathic epilepsy and owners' perspective on long-term management (5–9). Interestingly, around 50% of dog owners reported that their dog's seizures affected the ability to leave their dog unsupervised, afraid to miss a seizure (7) and 82% of dog owners, are reported to keep track of their dog's seizure activity (9). Although the gold standard for identifying seizures is the detection of ictal or inter-ictal electroencephalographic (EEG) abnormalities (10), this is impractical for long-term and real-life setting use (11). Seizure detection is currently done by owners' visual recognition. However, accurate recognition and recording of seizures has proven to be unreliable in people as half of the seizures recording during video-EEG are not known to the patient (12). This discrepancy between seizure recognition and seizure occurrence is also suspected to be true in dogs (13). Hence, there is a need to investigate other strategies in order to reliably measure response to treatment but also to improve the management of canine epilepsy for owners which in turn improves dog-owner connection. Wearable technology for dogs populates today's market with those that are commercially available mainly used for tracking location, however some claim use also for medical diagnosis and treatment (14).

Studies on wearable devices to detect seizures are extensively available in the human literature. These devices are based on detecting physiological changes before or during a seizure such as alterations in movement, heart rate and electrical activity in muscles (15). Considering the increasing interest and number of publications on these devices in human medicine, it would be interesting to investigate whether this could be translated to dogs. This review summarizes various seizure detection devices described in the humane literature, discusses their potential use and limitations in veterinary medicine and describes what is currently known in the veterinary literature.

## Seizure Detection Devices (SDD)

### Unimodal Parameters

Most investigated parameters include autonomic changes (cardiovascular, respiratory, and transpiration) and changes in movement (accelerometry, surface electromyography) (9). The devices this review will focus on, include heart rate (ECG), electrodermal activity (EDA), accelerometry (ACM), and surface electromyography (sEMG) (Table 1). The false alarm rate (FAR) of a device refers to the average number of times that the detector incorrectly declared the onset of seizures in a 24-h period (10). Should only a small proportion of the alarms be relevant to the caregiver (i.e., high false alarm rate), then the caregiver may stop responding to alarms, a phenomenon called “alarm fatigue.” In these cases, alarm fatigue may defeat the purpose of the device (11).

**Table 1:** ACM, accelerometer; CS, clonic seizure; ECG, electrocardiography; EDA, electrodermal activity; EMG, electromyography; FS, focal seizures; GTCS, generalized tonic–clonic seizure; HR, heart rate; NA, not available; Spo2, arterial oxygenation; TS, tonic seizure.

**Detection method**	**Article**	**Year of publication**	**Seizure type**	**False detection rate/24 h**	**Number of seizures**	**Reference standard**	**Sensitivity %**
**Unimodal non-EEG seizure detection parameters in generalized tonic and/or clonic seizure**							
ECG	De Cooman et al.	2017	FS and GTCS	47.28	127	Video-EEG	81.89
	De Cooman et al.	2020	Not specified.	45.6	227	Video-EEG	71
	Van Elmpt et al.	2006	TS and CS	NA	104	Video-EEG	>90
	Jeppesen et al.	2019	FS and GTCS	1	126	Video-EEG	93.1
	Jeppesen et al.	2020	GTCS, FS and non-convulsive seizures	0.22	48	Video-EEG	87
ACM	Becq et al.	2013	GTCS	NA	58	Video-EEG	90
	Beniczky et al.	2013	GTCS	0.2	39	Video-EEG	90
	Joo et al.	2017	GTCS	2	10	Video-EEG	100
	Kramer et al.	2011	TS, CS, GTCS	0.11	22	Video-EEG	91
	Lockman et al.	2011	GTCS	NA	8	Video-EEG	87.5
	Nijsen et al.	2010	TS	NA	64	Video	80
	Patterson et al.	2015	GTCS	NA	191	Video-EEG	31
	Meritam et al.	2018	GTCS	0.1	48	None	90
	Velez et al.	2016	GTCS	NA	62	Video-EEG	92.3
	Kusmaker et al.	2017	GTCS	0.72	21	Video-EEG	95.23
	Kusmaker et al.	2019	GTCS	0.7	46	None	95
sEMG	Beniczky et al.	2018	GTCS	0.7	32	Video-EEG	94
	Conradsen et al.	2012	GTCS	1	22	Video-EEG	100
	Halford et al.	2017	GTCS	1.14	46	Video-EEG	100
	Larsen et al.	2014	TS, CS	1.92–15.8	26	Video-EEG	100
	Szabo et al.	2015	FS and GTCS	NA	196	Video-EEG	95
**Multimodal non-EEG seizure detection parameters in generalized tonic and/or clonic seizure**							
ACM + EDA	Poh et al.	2012	GTCS	0.74	16	Video-EEG	94
ACM + sEMG	Milosevic et al.	2016	GTCS	0.56–1	22	Video-EEG	91
ECG + EDA + Spo2	Cogan et al.	2015	Not specified	0	7	EEG or none	100
ECG + Oximetry	Goldenholz et al.	2017	FS and TC	NA	193	Video-EEG	81
ACM + EDA	Onorati et al.	2017	GTCS	0.2	55	Video-EEG	95

### Heart Rate Variability (HRV)

Cardiac changes have been most extensively investigated before, during and after seizures as an extracerebral physiological parameter (12, 13). Cardiovascular changes are particularly important as they are linked to sudden unexplained death in epilepsy (SUDEP) in humans (15). Heart rate changes include tachycardia, bradycardia, and asystole. These changes are most prominent in generalized tonic-clonic type seizures, but unlike devices measuring movement, they can also detect non-convulsive seizures (16). Tachycardia is most consistently recorded during epileptic seizures and can be explained by an increased motor activity, release of catecholamines, sympathetic and parasympathetic shifts, activation of limbic structures, increased neuronal firing, or a combination of these and other unknown factors (17). Ictal bradycardia is less common and is more frequently associated with focal seizures (18, 19). Given the variable autonomic nervous system changes, heart rate variability (HRV) is preferred for the use of detecting seizures. In addition, there is also individual variation reflecting the unique individual spread and evolution of seizure activity (20, 21). This warrants the development of a patient-specific detection algorithm for HRV, and several proposals have been brought forward (8, 22). Most studies measuring HRV are retrospective validation studies (23–25). Sensitivity rates of 81.3–96.1% have been described, and a higher sensitivity has been associated with higher false alarms rate (up to 5.4/h). A prospective validation study was published recently which used a predefined detection algorithm based on heart HRV using patient-specific cut-off values. Responders were defined as patients who had a >50 beats/min ictal change in heart rate. The algorithm detected 9 out of 10 convulsive seizures with a false alarm rate of 0.9/24 h (0.22/night) (26). The study design, including video-EEG as long-term monitoring, and results are extremely promising and may form footing for further large-scale multicentre validation studies.

There are no veterinary studies investigating heart rate changes during the ictal phase. One study analyzed interictal HRV in presumed idiopathic epileptic dogs in comparison with non-epileptic dogs. Their findings revealed that dogs with idiopathic epilepsy were associated with an increased P wave dispersion QT interval, suggesting these dogs have cardiac electrical abnormalities similar to conductibility delays of the electric impulse observed in dogs with primary heart disease or electrolyte imbalances (27). Although these results cannot be directly used to detect seizures, the findings are interesting as such abnormalities are considered markers for severe arrhythmias associated with SUDEP in people (28).

### Electrodermal Activity (EDA)

Electrodermal activity (EDA), also known as skin conductance, reflects the activity of the sympathetic nerve on sweat glands (29). Epileptic seizures have shown to transiently increase EDA (30). This can be explained by the increased conductance of an applied current (such as by an EDA device) by sweat. Only a few studies have investigated EDA during the ictal and post-ictal phase. Their results showed that epileptic seizures induced a decrease in skin resistance by sweating. The epileptic seizures induce a surge in EDA and these changes are more prominent in GTCS which reflects a massive sympathetic discharge. The first long-term, video-EEG controlled study including seven patients, found that in GTCS, EDA increased in all (100%) patients by over 20 μS. In addition, EDA also remained significantly elevated for a longer time during GTCS compared to other types of seizures (31). The disadvantages of the use of EDA are the susceptibility to motion and pressure artifacts (32). Therefore, EDA used alone is associated with a high FAR and EDA is now increasingly used in combination with other non-EEG changes such as ACM (16).

Electrodermal activity has been used in a veterinary study to assess postoperative orthopedic pain (33). The product used in this study is currently off the market and there are no other studies investigating its use for detecting seizures. Also, sweat glands that actively participate in central thermoregulation in dogs are limited to the merocrine glands in the footpads (34). This is impractical for home use but could be considered in a hospital setting.

### Accelerometry (ACM)

Accelerometry (ACM) is the rate of change of velocity of the body in its own rest frame. It can be used to detect changes in velocity during a GTCS. Recent advances in technology have resulted in the development of ambulatory devices which are usually small, portable and easy to use (35, 36). An accelerometer has been proven to be able to detect a variety of seizures including focal seizures, GTCS and myoclonic, clonic, tonic, and hypermotor seizures (37). Initial studies used healthy subjects simulating a motor seizure, but this has evolved, and most studies nowadays use clinical subjects, a predefined or even patient-specific algorithm and video-EEG as long-term monitoring. More than a dozen studies have used accelerometers and most of them have implemented the ACM in a wrist-worn device (38). Three well-known commercially available human wrist-worn devices are the EpiWatch^®^, the SmartWatch^®^, and the Embrace^®^. The median sensitivity of the EpiWatch (Epi-Care, Danish Technology, Denmark) investigated by two studies was found to be 90% with a FAR of 0.1/day. The first study used video-EEG as seizure monitoring and the second study relied on seizure count by patient or caregiver as part of a field study (39, 40). The results of the SmartWatch were disappointing as they found a sensitivity of only 31% for detecting seizures using their device (SmartWatch, California, USA). This was a large prospective study using video-EEG as seizure monitoring and false alarms were not recorded in this study (41). The third device (Embrace, Boston, USA) was tested via a smaller, video-EEG controlled, prospective study. Sensitivity was 95% with a FAR of 0.7/day (42). Although sensitivity was high, there was noticeable inter-patient variation, and the sensitivity and specificity are dependent on the algorithm that was used for that patient.

Another recent wrist-worn device was developed within a non-commercial platform, and it evaluated three classification algorithms comparing them with video-EEG. The algorithm with the highest sensitivity for measuring tonic-clonic seizures was 100% with a FAR of 1.2/day and the algorithm with the lowest false positive rate had a sensitivity of 90% with a FAR 0.24/day (43), illustrating the influence of sensitivity on FAR. Non-convulsive movements were challenging to detect and detection also depends on the movement of the limb to which the device is attached. The use of the lower arm is recommended in humans, especially for capturing motor seizures (44). As a result, there is a risk to both over-and under detect seizures and future studies will focus on specific algorithms to increase sensitivity and specificity (16).

Similar problems have been encountered in veterinary medicine. A prospective single center study was performed to assess the accuracy of a collar-mounted accelerometer in dogs to detect seizure activity. The study consisted of two phases; a predefined algorithm was used to detect seizures during the first study phase, and an individualized algorithm was subsequently used during the second study phase. Seizures were manually recorded by owners. Both predefined and individualized algorithms had low sensitivities (18.6 and 22.1% respectively) to detect seizures with a low FAR (0.096/day and 0.054/day respectively). Reasons for these disappointing results could include the position of the device and the algorithms used in the study. The neck may not present such vigorous rhythmic movements during generalized tonic-clonic seizures, as seen for example in the limbs (45). However, long-term use of a device secured to the limb seems impractical in a dog with increased risk of device damage or ingestion.

### Surface Electromyography (sEMG)

Surface electromyography (sEMG) records muscle activity with as little as one channel and has emerged as a promising modality for detecting motor seizures in people (17, 46, 47). EMG signals provide direct information about electrical activity in the motor cortex as muscles are in direct synaptic contact with motor neurons. The mechanism of muscle activation has shown to differ between convulsive epileptic seizures and voluntary muscle activation as they show different electrographic patterns (16). The amplitude was for example found to be higher in patients with GTCS compared with patients with psychogenic non-epileptic seizures (PNES) and epileptic seizures had a larger duration in EMG activity (46). A recent prospective, video-EEG controlled, multicentre and blinded study used a relatively small device which could be worn under normal clothing and the device was attached by a self-adhesive hypoallergenic hydrogel patch. They found a high sensitivity (93.8%), short detection latency (9 s) and low number of false alarms (0.67/d) (47). Long-term recording of surface EMG activity is technically easy to perform, however disadvantages often are discomfort or skin irritation caused by the electrode patches (48). sEMG is an effective modality for the detection of seizures with a motor component but false negatives still occur, often triggered by common movements such as physical exercise (16).

Surface electromyography has been frequently used in veterinary medicine for analyzing muscle activity patterns during walking (49–52). As with EDA, no studies in veterinary medicine have focussed on the use of sEMG for detecting seizures. A possible advantage could be that the device can be placed almost anywhere on the body, for example the neck. A likely major disadvantage is the artifact of movement.

### Multimodal Parameters

A more recent development in human medicine is using a multimodal approach for detecting GTCS (38). Several wrist-worn devices have been developed combining non-EEG parameters. Results show that combining parameters improves sensitivity and lowers false-positive alarms by profiting from the strengths of each individual parameter (Table 1).

Most of these studies used accelerometers in combination with other parameters. A wristband measuring three-axis accelerometers and electrodermal activity yielded a high sensitivity (>92%) with a FAR of 0.2–1/day (42). Sensitivity and FAR improved during rest, still indicating the interference of non-convulsive movements. Another study found a sensitivity of 91% and a FAR of 0.2/day but was limited to nocturnal seizures only, which reduces the involvement of non-convulsive movements (53). Most recent studies are focused on detecting GTCS using multimodal parameters within a single device and using personalized algorithms (16).

## Clinical Validation of Seizure Detection Devices

Most studies investigating wearable seizure detection devices commercially available for people have focussed on overall performance including sensitivity and false alarm rate (FAR) (10). Despite the rapidly growing development of these devices, studies on accuracy remain scarce and the overall quality of the studies is low (9). Published studies have focussed on both motor and non-motor seizures, but their study designs are heterogenous and confusing (40, 49, 54). There are several shortcomings of device validation studies. Firstly, there is a lack of generalizability as there are studies with a small sample size, studies selecting highly specific patient populations and/or studies with a short study period. Some validation studies for example included <5 patients with recordings <5 days and others only included specific type of patients such as adult patients with impaired mental faculties. Second, most devices are tested in a hospital setting which enables the use of EEG to assess its performance. This probably does not reflect everyday ambulatory activity and using a mobile-EEG as control for outpatients would be recommended in these studies. Lastly, seizure type was not always specified and differentiation between day and night should be considered in case of self-reported seizure detection (55).

There are several systematic reviews available which have used a previously published general tool for systematic reviewing of diagnostic accuracy (35, 56). Beniczky and Ryvlin have recently attempted to standardize testing for validating specifically seizure detection devices, by providing a list of key features essential for studies on seizure detection. Depending on how these key features were incorporated, studies were classified into categories (phases) from 0 to 4, with 0 corresponding to initial studies on starting up or developing a new method, and 4 consisting of in-field studies of seizure detection devices in the home environment of the patients. The essential features of this system were grouped as subjects (simulated data or healthy subjects vs. low or high number of patients with seizures), recordings (conventional method vs. dedicated device, discontinuous vs. continuous, single center vs. multicentre, prospective vs. retrospective), analysis & alarm (training and testing using the dataset vs. predefined algorithm and cut off values, offline vs. real time, not blinded vs. blinded) and reference standard (none vs. video or video-EEG recordings) (35). But there are also technical factors that should be considered when measuring the accuracy and performance of a device, such as for example the device deficiency time (proportion of the time period when the device was not functioning), false alarm rate and detection latency (time in seconds from seizure onset of the targeted seizure to the time of seizure detection) (17, 57). So far, the best evidence to reliably detect a seizure in people, has been found for the seizure-type generalized tonic-clonic seizures (GTCS) (3). This is encouraging as this is the most frequently reported seizure type in dogs with idiopathic epilepsy (58, 59).

There are no published standards for study designs in the veterinary literature. Investigating non-EEG changes during the pre-ictal or ictal phase in dogs will require extensive research and, with the knowledge from human medicine, this should ideally be performed via a specific set of standards. Initial studies on starting up or developing a new method may include only a small number of subjects using a conventional or already existing device, and training and testing the algorithm will likely be done on the same data set without the use of the gold standard (video-EEG). If successful, this could lead to large prospective and real-time multicenter studies using predefined algorithms and cut-off values. Video-EEG as a long-term monitor would be the gold standard but due to its impracticality, EEG has never been established as a routine test in canine epilepsy in most referral centers (3). A modified version from the widely accepted human “international 10–20 system” for electrode scalp placement has been used but still no standardized EEG technique had been made in veterinary medicine although recently studies have been aiming to standardize EEG conditions (58–60). For now, veterinary studies continue to rely on visual information on seizure onset obtained in hospital or by the caregiver.

## Discussion

**Figure 1:**
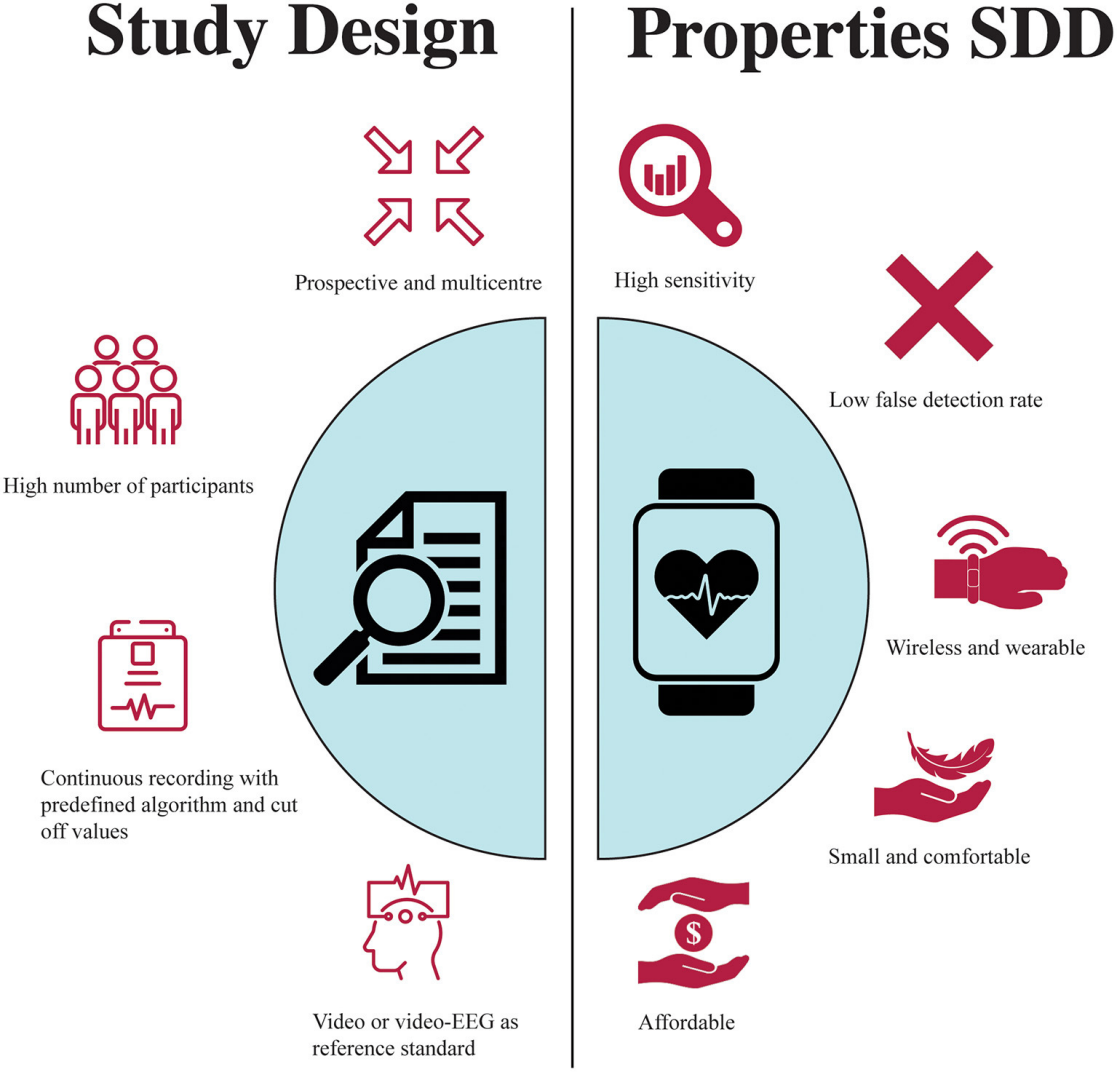
Key features Seizure Detection Device.

Monitoring dogs with epilepsy can be challenging for dog owners, but also for veterinarians and clinical staff managing hospitalised epileptic dogs. A reliable device that monitors and records seizures may help caregivers feel a sense of control, which may in turn reduce the burden of managing an epileptic dog. So far, there is only one non-EEG device tested in dogs, leaving many opportunities to further explore non-EEG seizure detection devices in veterinary medicine. User needs for dog owners appear similar to caregivers for people as for example they both value a high accuracy but would also accept a high false alarm rate (2, 61). Although the information on study design and device properties in humans is extensive and could potentially be translated to animals, there are many more challenges faced during the design of such a study in veterinary medicine. For example, deciding the anatomical location where the device should be placed and how to obtain a large enough sample size. Figure 1 summarises some of the key features for the study design and for the device itself. Nonetheless, given the challenges of EEG in veterinary medicine for seizure detection, using non-EEG parameters could be a promising alternative. Its use in dogs may help improve emergency intervention and improve hospitalization of epilepsy patients as they are currently kept for observation in an often-overstimulating ICU. If reliable, they may also provide a more accurate seizure count by not relying on the observations of an omnipresent carer for record keeping.

## Author Contributions

JB and CS: study conception and design. JB: draft manuscript preparation. CS and RG-Q: editing the manuscript. All authors revised the review and approved the final version of the manuscript.

## Conflict of Interest

The authors declare that the research was conducted in the absence of any commercial or financial relationships that could be construed as a potential conflict of interest.

## Publisher's Note

All claims expressed in this article are solely those of the authors and do not necessarily represent those of their affiliated organizations, or those of the publisher, the editors and the reviewers. Any product that may be evaluated in this article, or claim that may be made by its manufacturer, is not guaranteed or endorsed by the publisher.
